# Comparative genomics of cetartiodactyla: energy metabolism underpins the transition to an aquatic lifestyle

**DOI:** 10.1093/conphys/coaa136

**Published:** 2021-01-16

**Authors:** Davina Derous, Jagajjit Sahu, Alex Douglas, David Lusseau, Marius Wenzel

**Affiliations:** 1 School of Biological Sciences, University of Aberdeen, Aberdeen, UK; 2 National Institute of Aquatic Resources, Technical University of Denmark, Kgs. Lyngby, Copenhagen, Denmark; 3 Centre for Genome Enabled Biology and Medicine, University of Aberdeen, Aberdeen, UK

**Keywords:** Cetaceans, energy metabolism, evolution, insulin, mTOR, NF-ĸB

## Abstract

Foraging disruption caused by human activities is emerging as a key issue in cetacean conservation because it can affect nutrient levels and the amount of energy available to individuals to invest into reproduction. Our ability to predict how anthropogenic stressors affect these ecological processes and ultimately population trajectory depends crucially on our understanding of the complex physiological mechanisms that detect nutrient availability and regulate energy metabolism, foraging behavior and life-history decisions. These physiological mechanisms are likely to differ considerably from terrestrial mammalian model systems. Here, we examine nucleotide substitution rates in cetacean and other artiodactyl genomes to identify signatures of selection in genes associated with nutrient sensing pathways. We also estimated the likely physiological consequences of adaptive amino acid substitutions for pathway functions. Our results highlight that genes involved in the insulin, mTOR and NF-ĸB pathways are subject to significant positive selection in cetaceans compared to terrestrial artiodactyla. These genes may have been positively selected to enable cetaceans to adapt to a glucose-poor diet, to overcome deleterious effects caused by hypoxia during diving (e.g. oxidative stress and inflammation) and to modify fat-depot signaling functions in a manner different to terrestrial mammals. We thus show that adaptation in cetaceans to an aquatic lifestyle significantly affected functions in nutrient sensing pathways. The use of fat stores as a condition index in cetaceans may be confounded by the multiple and critical roles fat has in regulating cetacean metabolism, foraging behavior and diving physiology.

## Teaser

Anthropogenic stressors can disrupt cetacean foraging ability and reduce energy available for reproduction. Predicting such ecological consequences is limited by our understanding of cetacean energy metabolism. We here show that cetaceans have undergone substantial evolutionary changes in nutrient signaling metabolic pathways, rendering mechanistic physiological frameworks derived from model organisms inappropriate.

## Introduction

Sub-lethal anthropogenic stressors (e.g. noise, contaminants or prey limitation) are becoming a pervasive and prevalent threat to many cetacean species and are a key priority in cetacean conservation policy (e.g. National Academies of Sciences, 2017). One important, though often overlooked, issue is the extent to which anthropogenic disturbances can perturb environmental nutrient levels and thus affect cetacean foraging abilities. For example, anthropogenic noise caused by pinniped acoustic deterrent devices can indirectly impact harbor porpoises (*Phocoena phocoena*) by causing displacement from important habitat and feeding grounds (Simonis *et al.,* 2020). This will lead to disrupted foraging, increased stress and impacts on the amount of energy invested in reproduction, which will ultimately impact the population trajectory (Simonis *et al.,* 2020). In addition, sound produced by naval activity and exercises can impact behavior of marine species and mass strandings have occurred in coincident with the presence of naval vessels (Bernaldo de Quirós *et al.,* 2019). Our ability to understand the effects of anthropogenic stressors on cetacean ecology depends crucially on our understanding of the complex physiological signaling mechanisms that detect nutrient availability and regulate energy metabolism, foraging behavior and life-history decisions.

One significant barrier to achieving this understanding is that these physiological mechanisms are likely to differ considerably from those of terrestrial mammal model systems. Cetaceans evolved from terrestrial mammals approximately 53–56 million years ago and drastically adapted their anatomy, physiology and metabolism during transition to an aquatic lifestyle. In particular, the ability to maintain body temperature and energy reserves required several critical morphological and physiological adaptations (Scholander *et al.,* 1950). For example, a thickening of the blubber was essential to provide thermal insulation and to store energy to compensate for more sporadic foraging opportunities (Williams *et al.,* 1993, 2001; Vasseur and Yodzis, 2004). Blubber tissue also produces the hormone leptin, which plays a crucial role in signalling energy status to other organs involved in regulating energy metabolism. In model organisms such as humans and laboratory rodents, leptin secretion is proportional to the amount of adipose tissue and also plays a role in mammalian reproduction whereby both low and abnormally high energy stores lead to reduced reproductive output (Caprio *et al.,* 2001). In cetaceans, leptin levels show unusually large seasonal variation and are associated with physiological and behavioral changes (e.g. the onset of migration) (Ball *et al.,* 2017). Therefore, leptin may not be a measure of total lipid stores in cetaceans to the same degree as we would expect in terrestrial mammals (Ball *et al.,* 2017). This means that regulation of energy metabolism in cetaceans may depart from the classical mammalian model and temporary leptin resistance during migration may be a mechanism to reduce the anorexigenic effects of leptin (Ball *et al.,* 2017).

An intriguing consequence of this is that increased adiposity in cetaceans is unlikely to have the same deleterious health consequences (e.g. obesity) as in terrestrial mammals such as humans and laboratory rodent models. In these models, both reduced and increased fat mass (signaled by leptin) lead to reduced reproductive success (Caprio *et al.,* 2001), while pregnancy rate in North Atlantic fin whales (*Balaenoptera physalus*) is impacted by lower but not larger than normal blubber thickness (Williams *et al.,* 2013). Hence, cetaceans may be less constrained on the amount of fat they are able to store compared to humans and other terrestrial mammals. However, there may be physiological constraints imposed by the cost to overcome blubber buoyancy during dives (Nowacek *et al.,* 2001; Miller *et al.,* 2004, 2012). In fact, diving strategy is directly affected by blubber thickness and is optimized to reduce energy expenditure. For example, North Atlantic right whales (*Eubalaena glacialis)* with lower amounts of blubber angle their body in such a way to reduce the cost of transport when swimming against the force of buoyancy (Nousek-McGregor *et al.,* 2014). This may indicate that during periods of nutritional stress (e.g. migration), the energetic cost of swimming is too high and requires adjustment in behavior to preserve energy.

These complex trade-offs between blubber thickness, buoyancy, energy expenditure during diving, foraging and migration behavior and reproductive investment are likely to be influenced by nutrient availability, which in turn is impacted by environmental pressures such as anthropogenic stressors. We here take the view that the evolutionary transition from a terrestrial to an aquatic lifestyle necessitated complex cellular and physiological changes in nutrient sensing mechanisms that catalysed downstream metabolic and behavioral adaptations for resolving life-history trade-offs based on energetic state. We test this view with a comparative genomics approach to identifying signatures of natural selection in cetacean lineages contrasted with terrestrial mammals, focusing specifically on nutrient signaling pathways. This targeted approach allows us to examine how evolutionary changes in these pathways affect energy metabolism and how this may differ from the classic mammalian model. These insights are important for understanding how conservation efforts may have to be focused on managing environmental stressors that impact nutrient availability.

We focus on six key metabolic pathways involved in signaling nutrient availability in model organisms: leptin signaling, insulin signaling, p53 signaling, mTOR signaling, SIRT (Sirtuin) signaling and NF-ĸB signaling. Leptin and insulin represent the classic model feedback system that can lead to changes in behavioral and metabolic responses in mammals (Obici and Rossetti, 2003). However, previous work has not found any evidence that leptin signaling itself is functionally different in cetaceans but speculated that its downstream pathways might be (Ball *et al.,* 2017). In response to limited nutrient availability the p53 signaling pathway can downregulate AKT/mTOR pathways (Puzio-Kuter, 2011), which in turn allows for more nutrient transporters to be expressed to the cell surface and increases uptake of nutrients (e.g. glucose and amino acids). Sirtuin 1 can directly sense the metabolic status of a cell and regulate metabolic processes related to stress and energy metabolism (Li, 2013). Finally, as cetaceans may not face the same metabolic pathologies associated with obesity in humans and laboratory rodents, we also targeted NF-ĸB signaling pathway. This pathway is one of the key mechanisms to signal inflammation caused by increased adiposity, which is associated with metabolic diseases (Baker *et al.,* 2011). We hypothesized that, instead of altering functionality of the signal (e.g. leptin), the target receptors and downstream pathway may be altered, having implications on how these signals may be transferred biologically.

## Material and Methods

### Focal pathways, genes and genomes

We identified 532 human reference genes involved in the leptin, insulin, p53, mTOR, SIRT or NF-ĸB signaling pathways using the Kyoto Encyclopedia of Genes and Genomes website (http://www.genome.jp/kegg/) and the ingenuity pathway analysis (IPA) program (version 2000–2019, Ingenuity Systems, www.ingenuity.com). We obtained reference genome assemblies for human, mouse, 16 cetacean species and 37 artiodactyl species from NCBI (Table S1). Since only 22 of these 55 genomes had protein annotations available, we identified gene homologues in all genomes using the human genes as a reference. The full amino-acid sequences of the genes were aligned to each genome using *EXONERATE* 2.2.0 (Slater and Birney, 2005) with the *protein2genome* model to obtain correct gene models with spliced alignments across introns. The single best alignment for each protein (maximum alignment score) was retained and the nucleotide sequence of the aligned genomic region was extracted.

Although this conservative approach potentially misses information contained in alternative isoforms, paralogues and pseudogenes, it provides a confident most representative match most similar to the human reference protein. For most proteins, the best match had a vastly higher alignment score than other matches, leaving little doubt over its accuracy. However, in other cases, we observed additional matches with the same alignment length as the best match and slightly worse alignment scores, suggesting the presence of alternative gene copies with sequence polymorphisms. We identified 61 of 532 proteins where more than half of the cetacean genomes were affected; we noted these proteins as potentially problematic throughout our analyses, but none of them showed signatures of selection and thus they did not cause us to make false positive conclusions.

### Identification of positive selection on genes and amino-acid substitutions

For each of the 532 genes, all homologues obtained from the 55 genomes were codon-aligned with guidance from translated amino-acid alignments and allowing for frame shifts using *MACSE* 2.03 (Ranwez *et al.,* 2018). Maximum-likelihood gene trees were inferred from these alignments using *IQTREE* 1.6.8 (Nguyen *et al.,* 2015) with automatic selection of nucleotide substitution models. A consensus species tree was then constructed from all 532 gene trees using *ASTRAL-III* 5.6.3 (Zhang *et al.,* 2018) and rooted at the two outgroups, human and mouse.

To identify signatures of positive selection, codon sequence evolution was modelled in the *codeml* program of *PAML* 4.9f (Yang, 2007), using the inferred species tree with a trifurcated root (=derooted) as a reference. Briefly, this approach is based on comparing the ratio of non-synonymous mutations (i.e. mutations that cause a change in amino acid) and synonymous mutations (i.e. mutations that do not change the amino acid) across genes, codons and tree branches. This dN:dS ratio (ω) is expected to be 1 under neutral evolution (all mutations are equally likely), smaller than 1 under purifying selection (non-synonymous mutations are purged) and greater than 1 under positive selection (non-synonymous mutations are beneficial) (Yang, 2007). By fitting a number of models that allow ω to vary heterogeneously among codons and/or branches, the most likely evolutionary model can be identified with likelihood-ratio tests (e.g. Areal *et al.,* 2011; Yao *et al.,* 2019).

To minimize the impact of gaps in the alignment, all codons with more than 20% missing data were removed from the alignments using *trimal* 1.4 (Capella-Gutiérrez *et al.,* 2009). Three models were run per alignment. First, the null model estimated a single dN:dS ratio (ω) for the entire alignment. Second, the branch model (model = 2; NSsites = 0) estimated a single ω for all cetacean lineages (foreground) and a different ω for all other lineages (background). This model thus assumes evolutionary change occurring solely in the cetacean phylogenetic group and across the entire protein. Third, the branch-site model (model = 2; NSsites = 2) estimated different ω ratios among codons within the foreground and background branches. This most realistic model thus assumes episodic evolutionary change in the cetacean branch at a subset of codons across the protein (Gharib and Robinson-Rechavi, 2013). The branch models and branch-site models were each run twice, first estimating foreground ω and second fixing ω at 1 (enforcing neutral evolution). The statistical significance of the ω estimates was obtained via likelihood-ratio tests carried out in *R* 3.4.0 (R Core Team, 2015). Both the free branch model and the positive branch-site model were contrasted with their corresponding neutral models (ω = 1) and with the null model by comparing twice the difference in likelihood (2ΔL) of the models against a Chi-square distribution with one degree of freedom. *P* values were corrected for multiple testing across all genes within each type of contrast using the false-discovery-rate method (Benjamini and Hochberg, 1995).

For all genes where the positive branch-site model fitted significantly better than the neutral branch-site model and the null model (i.e. evidence of positive selection), we identified the specific codons under positive selection in the cetacean lineage using the Bayes Empirical Bayes (BEB) method (Yang, 2007). We then examined whether the observed amino-acid substitutions in the cetacean lineage at these codons could have impacts on the biological function of the protein, using *PROVEAN* 1.1.5 (Choi *et al.,* 2012). This method measures the differences in sequence alignment score (from BLASTP) of a query protein sequence to similar sequences on NCBI before and after the amino-acid variant is introduced. If the variant reduces the alignment score consistently (ΔQ ≤ −2.5), a functional change is inferred. Since *PROVEAN* is primarily used to examine disease-causing variants in humans where the reference variant is known to be beneficial, changes in alignment score are usually interpreted as being deleterious to the function of the protein (Choi *et al.,* 2012). However, the statistical analysis of alignment scores underlying ΔQ is entirely generic and makes no assumptions of deleteriousness or benefit of variants (Choi *et al.,* 2012). Therefore, ΔQ ≤ −2.5 may equally be interpreted as a beneficial change in protein function consistent with positive selection. For each codon with BEB evidence of positive selection, we thus queried the most frequently observed amino acid among the 16 cetacean genomes against the *Homo sapiens* reference sequence; this design directly tests for functional changes in cetacea compared to the human outgroup.

### Pathway-level functional effects

We examined the identified signatures of positive selection among the 532 individual genes on a pathway level using IPA (version 2000–2019, Ingenuity Systems, www.ingenuity.com). This software allows for visualizing and analysing prebuilt biological pathways using measured values at particular genes. The links between the genes in IPA are knowledge based and therefore represent regulatory roles with either activator or inhibitor effects. This allows for predicting the regulatory impact a gene may have on the pathway. We used pre-build pathways for the p53 signaling pathway, the insulin signaling pathway, the mTOR signaling pathway, the leptin signaling pathway and the NF-ĸB signaling pathway. IPA did not have a prebuilt sirtuin signaling pathway, so we manually constructed this pathway based on the summarized data by (Nakagawa and Guarente, 2011).

We first visualized the interactions of genes under positive selection at a pathway level using IPA signaling pathways for each of the target pathways. The value of statistically significant likelihood-ratio test statistics (2ΔL) between the positive and neutral branch-site models was used as a measure for visualization. This allowed us to see how genes under positive selection may interact with each other and what the potential impact the gene may have on a pathway level.

We then assessed the pathway-level impacts of those genes that were identified with a functionally relevant amino acid substitution (PROVEAN score ≤ −2.5), using the molecule activity predictor (MAP) function in IPA. This function can predict the upstream (i.e. higher up in the signaling pathway) or downstream (i.e. lower in the signaling pathway) effect of a molecule in a pathway, allowing us to examine how these genes may impact energy metabolism by simulating directional consequences on downstream molecules and inferred activity upstream from the gene. The algorithm of the MAP function divides the molecules of the pathway into two sets: known or unknown expression based on the uploaded dataset. In our case, we used the PROVEAN score as a measure of ‘expression’. The algorithm identifies the unknown molecules that are connected to the known molecules by directional relationships (i.e. activated or inhibited) based on the underlying scientific knowledge (pre-existing content in IPA). For each molecule, it will then calculate a z-score using the findings between the unknown molecules and its known neighbors. Once the value of a molecule is known, it is moved to the ‘known set’ and the algorithm is repeated until all molecules have been evaluated. The result of the MAP function is the visualized predicted regulation of the pathway based on the genes affected by the functionally relevant amino acid substitution. Although this approach assumes that the identified beneficial amino-acid substitutions cause a concomitant change in protein expression, it provides a reasonable functional representation of those parts of the pathway that may be affected downstream due to functionally relevant amino acid substitution of any gene higher up in the signaling cascade.

## Results

### Signatures of selection in genes involved in nutrient sensing pathways

**Figure 1:**
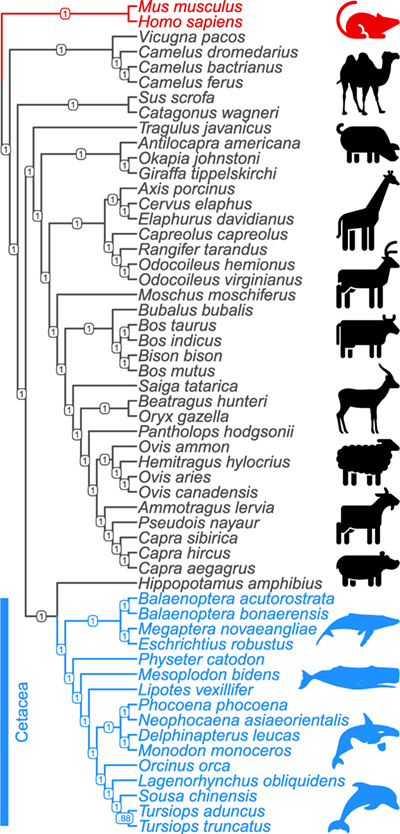
Species cladogram of the cetacea ingroup (blue) and artiodactyla, human and mouse outgroups (black and red), derived from 532 genes selected from six key nutrient-sensing pathways. Branch labels display posterior branch probabilities.

**Figure 2:**
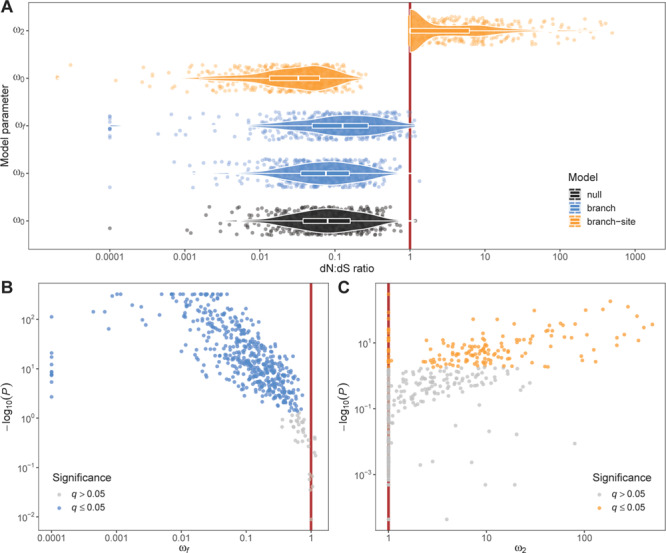
Ratios of non-synonymous versus synonymous nucleotide substitution rates (dN:dS ratios; ω) in 532 genes estimated from codon evolution models in PAML. (A) baseline estimates for whole alignments from null models (ω_0_), estimates for foreground (cetacea; ω_f_) and background (all others; ω_b_) branches from branch models, and estimates for codons under purifying (ω_0_) and positive (ω_2_) selection in foreground branch (cetacea) from branch-site models. (B) Foreground dN:dS ratio (ω_f_) and statistical significance (*P* value) from likelihood-ratio tests between free-ratio branch models and neutral branch models. Significant tests after FDR correction (q ≤ 0.05) are highlighted in blue. (C) Foreground dN:dS ratio of positively selected codons (ω_2_) and statistical significance (*P* value) from likelihood-ratio tests between positive-selection branch-site models and neutral branch-site models. Significant tests after FDR correction (q ≤ 0.05) are highlighted in orange. The red solid lines in all plots represent neutral evolution (ω = 1).

The species tree among 16 cetacean species and 37 artiodactyl species based on 532 gene trees placed the cetacean clade as sister group to *Hippopotamus amphibius* (Fig. 1), consistent with published cetartiodactylan phylogenies (Zurano *et al.*, 2019). Based on this tree, gene-level estimates of dN:dS ratio (ω_0_) from the PAML null models were < 1 for all but one genes (median ω_0_: 0.08; mean ω_0_: 0.12), consistent with an expected baseline of strong purifying selection on protein function across all taxa and all codons (Fig. 2A). Branch models that contrasted the cetacean group with all other taxa revealed that 224 of 532 genes (42.1%) departed significantly (FDR-corrected q ≤ 0.05) from neutral codon evolution (ω ≠ 1), but all of these genes were under purifying selection (ω_f_ < 1) instead of positive selection (ω_f_ > 1). Only 7 of 532 genes (1.3%) showed an indication of positive selection (ω_f_ > 1), but none of these estimates were significant (Fig. 2B). In contrast, branch-site models revealed significant (q ≤ 0.05) positive selection (median ω_2_: 9.67; mean ω_2_: 39.64) in the cetacean group on a subset of codons in 133 of 532 genes (25%) (Fig. 2C).

### Functional effects of amino-acid substitutions

**Figure 3:**
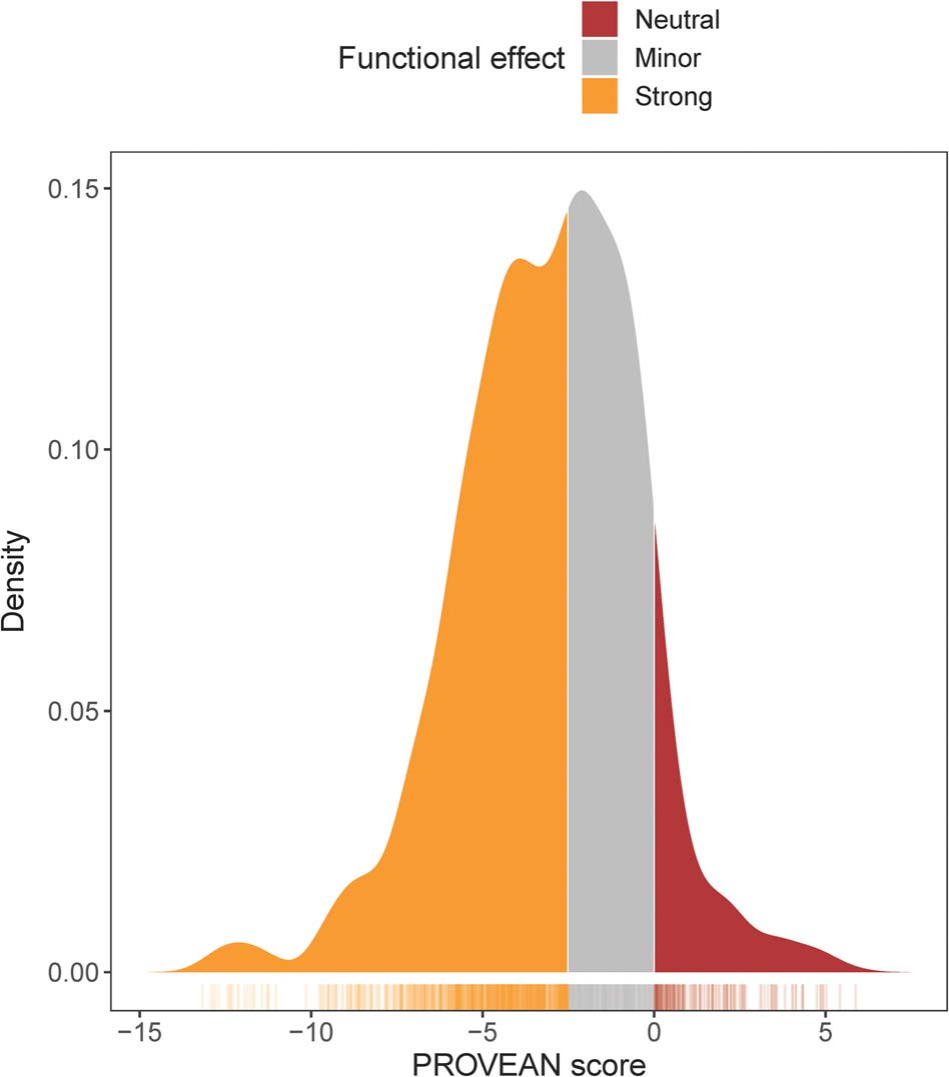
Distribution of PROVEAN scores of 1936 amino-acid substitutions under positive selection in cetacea. Functional effects of substitutions are colour-coded according to standard PROVEAN thresholds: positive scores indicate neutral effects and scores below −2.5 indicate biologically significant functional changes.

Using the BEB method in PAML, a total of 1936 codons among 133 genes (mean 14.78 codons per gene) were under positive selection in the cetacean lineage. The vast majority (91%) of amino-acid substitutions in cetacea were predicted to have some functional effects (PROVEAN score < 0) and 56% of substitutions may have strong, biologically significant, effects (PROVEAN scores ≤ −2.5; median score: −2.9). Only 8.5% of substitutions had a positive PROVEAN score, suggesting functional neutrality (Fig. 3).

### Pathway-level interactions among genes under positive selection

Using IPA to visualize genes under positive selection in cetacea at the pathway level, we found evidence of selection in insulin signaling (Fig. S1, Table S2), mTOR signaling (Fig. S2, Table S3), NF-ĸB signaling (Fig. S3, Table S4) and SIRT signaling (Fig. S4, Table S5). A large number of genes under selection were related to glucose metabolism and inflammation. We also highlight genes upstream from lipid metabolism, cell growth and proliferation and apoptosis functions to be under positive selection. In contrast, little evidence of selection was found for p53 signaling (Fig. S5, Table S6) and leptin signaling (Fig. S6, Table S7).

**Figure 4:**
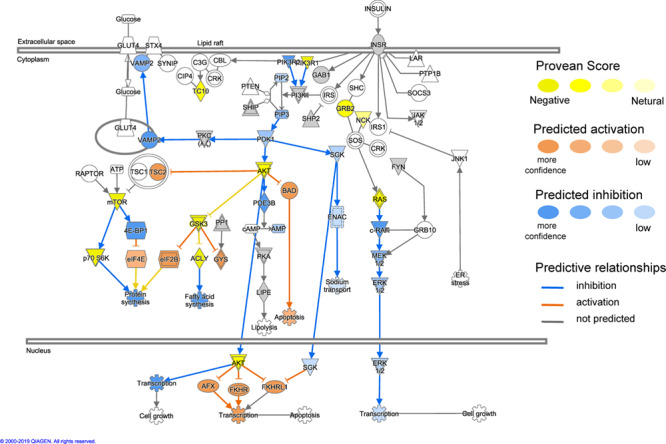
Functional interactions between genes under positive selection in the insulin signaling pathway (ingenuity pathway analysis, IPA). Genes with functionally relevant amino-acid substitutions (PROVEAN score ≤ −2.5) coloured in yellow. Possible downstream effects of these genes were visualized using the molecule activity predictor (MAP) tool in IPA (see prediction legend). The interactions highlight evolutionary changes in glucose metabolism.

**Figure 5:**
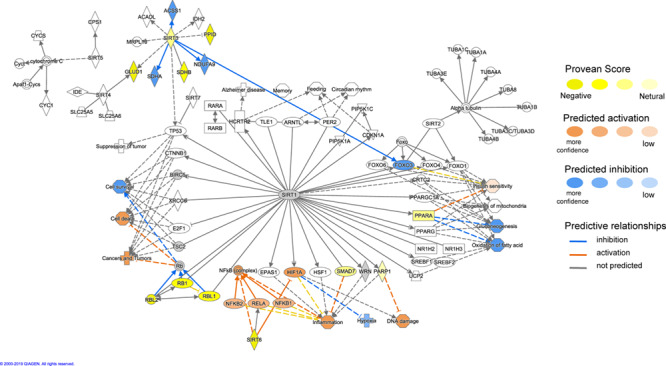
Functional interactions between genes under positive selection in the SIRT signaling pathway (ingenuity pathway analysis, IPA). Genes with functionally relevant amino-acid substitutions (PROVEAN score ≤ −2.5) coloured in yellow. Possible downstream damaging effects of these genes were visualized using the molecule activity predictor (MAP) tool in IPA (see prediction legend). These highlight changes to gluconeogenesis and fatty acid oxidation.

**Figure 6:**
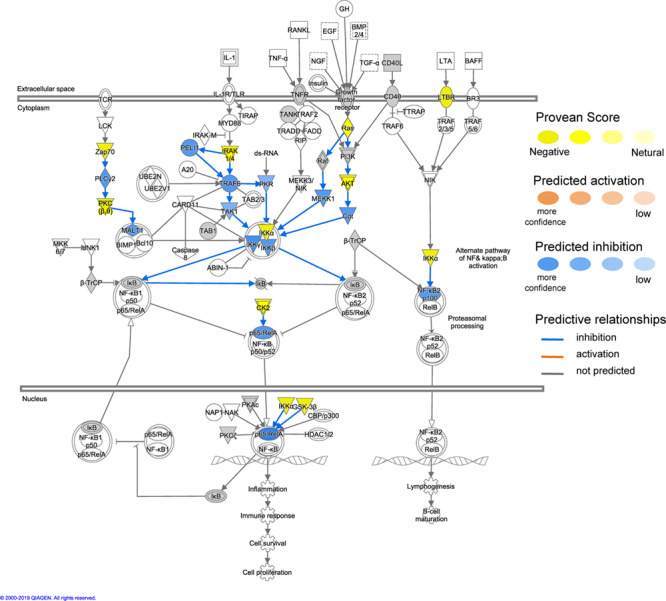
Functional interactions between genes under positive selection in the NF-ĸB signaling pathway (ingenuity pathway analysis, IPA). Genes with functionally relevant amino-acid substitutions (PROVEAN score ≤ −2.5) coloured in yellow. Possible downstream damaging effects of these genes were visualized using the molecule activity predictor (MAP) tool in IPA (see prediction legend). These highlight changes in responses to hypoxia and inflammation.

**Figure 7:**
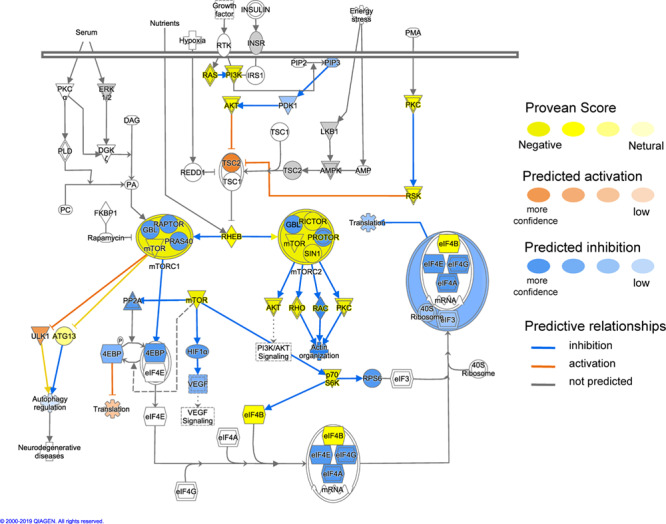
Functional interactions between genes under positive selection in the mTOR signaling pathway (ingenuity pathway analysis, IPA). Genes with functionally relevant amino-acid substitutions (PROVEAN score ≤ −2.5) coloured in yellow. Possible downstream damaging effects of these genes were visualized using the molecule activity predictor (MAP) tool in IPA (see prediction legend). These highlight important changes to key biological decisions about energetic investment.

Using the MAP function in IPA to predict potential pathway-level regulatory effects of functionally relevant amino acid substitutions identified by PROVEAN, we found several instances of directional consequences on downstream molecules and inferred activity upstream of the gene in the pathway. Unsurprisingly, glucose metabolism is expected to be altered with changes in insulin signaling (Fig. 4), SIRT3 signaling with downstream regulation of insulin sensitivity, PPARA signaling with downstream regulation of gluconeogenesis and oxidation of fatty acids based on the predicted directional consequences of the genes (Fig. 5). In addition, we expect changes in upstream signaling of inflammation, hypoxia and cell survival via SIRT6, RB1, NFKB and HIF1a (Figs 5 and 6). Most importantly, both the mTOR complexes and their upstream and downstream genes were identified to be differentially regulated (Fig. 7). These regulatory changes are expected to have effects on nutrient sensing and protein synthesis as well as key biological decisions about energetic investment such as shift to glycolysis and *de novo* lipid synthesis.

## Discussion

Sublethal stressors caused by anthropogenic disturbances can trigger a stress response regulated via the hypothalamic–pituitary–adrenal axis and signals to release energy in the form of glucose from glycogen stores or amino acids (gluconeogenesis) and from fat stores to allow a rapid response to optimize survival (i.e. fight and flight response) (Khani and Tayek, 2001). Cetaceans have a typical mammalian response to acute stress (Fair *et al.,* 2014; Champagne *et al.,* 2018); however, they may have crucial evolutionary changes to its energy metabolism, which may impact the detrimental effects that these stressors could have. Anthropogenic sublethal stressors can also reduce energy intake by changing motivational states resulting in a deprioritization of foraging. Together, these stressors may deplete the energy stores of affected individuals to such a degree that the energetic cost of reproduction cannot be met and would lead to a decline in population growth rate and health (Deyarmin *et al.,* 2019). We therefore need to move towards a greater understanding of how cetaceans signal energy status and regulate their energy metabolism.

There are indications that cetaceans underwent evolutionary adaptation to key genes involved in energy metabolism and this may impact the ways in which cetaceans cope with varied environmental pressures (Wang *et al.,* 2015; Endo *et al.,* 2018). Here, we took a targeted approach to identify positive selected genes in six nutrient sensing pathways. This allowed us to better understand how combined changes might be focused on particular functions within these pathways and what the physiological consequences of these changes might be for the way by which energy metabolism in cetaceans may differ from their terrestrial counterparts. These pathways have signaling cascades in common with hormones such as insulin and are linked with the release of hormones from adipose tissue (e.g. leptin). Our results indicate that genes involved in the insulin signaling pathway, the mTOR, SIRT and NF-ĸB signaling pathway were significantly positively selected. These pathways have profound effects on metabolism and the maintenance of energy reserves. The positive selection of genes related to glucose metabolism and inflammation suggests that these genes may have been positively selected to adapt to a glucose-poor diet and that fat deposits signaling may not be as limited by inflammation and metabolic dysfunctions (e.g. insulin resistance). Understanding these adaptations can help us identify how wild cetaceans could metabolically respond to multiple stressors and can help us manage conservation threats that perturb the environmental nutrient levels of cetaceans (*National Academies of Sciences*, 2017).

### Changes in glucose metabolism in response to a glucose-poor heterogeneous prey field

Cetaceans have a diet with a high fat and protein content and are almost devoid of carbohydrates (Wells *et al.,* 2013). Hence pathways regulating carbohydrate and glucose metabolism would have been under selective pressure as these species underwent a shift in substrate utilization. Genes related to the control of food intake, glycerol uptake, glucose metabolism and lipid metabolism were found to be under positive selective pressure in dolphins (McGowen *et al.,* 2012; Wang *et al.,* 2015). This is reflected in fasting studies with bottlenose dolphins (*Tursiops truncatus*) where glucose levels were significantly higher during starvation (Venn-Watson and Ridgway, 2007). In addition, fed a big meal of fish, bottlenose dolphins show an increase in insulin levels but when fed just dextrose and water, no increase in circulating insulin levels was observed (Venn-Watson *et al.,* 2013). This is surprising as insulin increases glucose transport via a signaling cascade to insert more glucose transporters (GLUT4) into the cell membrane, mainly in fat and muscle (Stöckli *et al.,* 2011). As glucose sources in cetaceans’ prey are limited, glucose transport may be prioritized for erythrocytes and specific brain regions under normal physiological or stressful conditions (Craik *et al.,* 1998). Hence, these studies suggest selection for insulin resistance in cetaceans. We found positive selections in the insulin signaling pathway, which are consistent with this hypothesis. In our study, both phosphatidylinositol 3-kinase (PI3K) and phosphorylated insulin receptor substrate 1 (IRS1) genes were estimated to be under positive selection but not GLUT4. We indeed identified functionally relevant changes to the insulin signaling pathway, including the Akt protein signaling kinase (Akt) and PI3K. Damage to this pathway in various tissues has been linked to insulin resistance in model organisms (Huang *et al.,* 2018). In fasted Northern elephant seals (*Mirounga angustirostris*), components of the insulin signaling pathway were reduced including PI3K and IRS1 (Viscarra *et al.,* 2011). Elephant seals are also insulin resistant (Viscarra *et al.,* 2011) and hence may share some common evolutionary selection of those mechanisms with cetaceans. Interestingly, glycogen made little contribution as an energy source during the 4-month fasting period in northern elephant seals but their fat as a source does and indeed evidence suggests that these animals differ from a ‘classic’ insulin response for substrate metabolism (Houser *et al.,* 2007; Champagne *et al.,* 2012). These fasted adapted animals may show some commonalities with migratory cetaceans where feeding is very sporadic (i.e. fasting state) and they rely on their lipid stores (George *et al.,* 1989; Ball *et al.,* 2015). Hence, insulin resistance or changes to the GLUT4-insulin cascade could potentially prevent glucose uptake by those tissues that can utilize fat as a source to prioritize the brain and red blood cells. Hence, in cetaceans, insulin resistance may be an evolutionary advantage to cope with the more sporadic feeding opportunities and migratory-starvation period in some species. This shows similarities with the results by Ball *et al.,* 2017, where leptin and its receptor were highly conserved and the difference in leptin function might stem from changes to its regulatory mechanism. Temporary leptin resistance in these animals may be a mechanism to induce a physiological response to change feeding habits or start migration (Ball *et al.,* 2017). This is in stark contrast with model organisms and humans where leptin secretion is in proportion to the amount of adipose tissue and serves as a signal for total lipids stores to communicate energy status and is linked to reproduction (Caprio *et al.,* 2001; Friedman, 2019).

### Thermoregulatory needs for adiposity and its implications for cellular hypoxia and inflammation

A large volume of adipose tissue triggers inflammatory responses in humans and laboratory rodents and can lead to metabolic dysfunctions at a physiological level (e.g. insulin resistance) (Mantovani *et al.,* 2002). In these mammals, adipose tissue can be categorized into two main types based on the position: visceral and subcutaneous fat. Expansion of visceral fat is associated with numerous metabolic disorders including insulin resistance and cardiovascular diseases (Mathieu *et al.,* 2010). NF-ĸB is involved in the molecular signaling of this adipose tissue expansion and triggers inflammatory responses (Ye *et al.,* 2007). As the inflammatory function of NF-ĸB is linked to the amount of fat mass, the high level of adiposity in cetaceans would in theory lead to chronic inflammation. The thickened blubber of cetaceans is a result of the secondary adaptation to life in water and hence selective pressure in this pathway may be a way to reduce intrinsic tissue inflammation. Here, we found that key genes in the NF-ĸB signaling pathway were positively selected inhibitor of nuclear factor kappa B kinase subunit beta (IKKβ) and IKKα. Mice that have the inflammatory pathway of NF-ĸB disabled (IKKβ knockout) are more insulin sensitive and are partially protected from high fat diet induced glucose intolerance and hyperinsulinemia (Arkan *et al.,* 2005). In addition, the gene regulation of receptor interacting serine/threonine kinase 1 (RIPK1) was also positively selected and its associated amino acid sequence changed drastically compared to the outgroups. RIPK1 has a downstream effect on IKKα and IKKβ, and hence may influence the signaling in this pathway. The IKK complex has a NF-ĸB independent role in the protection of cells from RIPK-dependent death downstream from the tumor necrosis factor rector (TNFR1) (Dondelinger *et al.,* 2015). It has to be noted that the thick blubber layer in cetaceans is classified as subcutaneous adipose fat and in contrast to visceral fat, expansion of subcutaneous fat has no detrimental effects on health (Kim *et al.,* 2007). Blubber is, however, metabolically active with expression of proteins involved in metabolism, immune response, inflammation and lipid metabolism (Kershaw *et al.,* 2018). In murine starvation studies where visceral fat is being used up as an energy source, subcutaneous fat changed its morphological and molecular characteristics to resemble visceral fat (Ding *et al.,* 2016). Hence, blubber may indeed have adapted those characteristics to maintain the signaling role of adipose tissue in whole body metabolism. However, it is unclear at this point to what degree blubber shares characteristics with visceral fat and if indeed selective pressure in NF-ĸB pathway may be a way to reduce intrinsic tissue inflammation. In addition, blubber can be divided in different layers and each layer seems to serve a different function (Hashimoto *et al.,* 2015). Further research into the metabolic functioning of blubber is needed to develop appropriate markers to assess nutritional condition and health in free ranging cetaceans (Kershaw *et al.,* 2019; Derous *et al.,* 2020).

In addition, NF-ĸB signaling is also linked to oxidative stress-induced inflammation (Lingappan, 2018). During dives, cetaceans experience a hypoxemia state (i.e. low oxygen) (Allen and Vázquez-Medina, 2019). Acute hypoxia can lead to a decrease in ATP synthesis via the mitochondrial oxidative phosphorylation and this will increase the production of reactive oxygen species (Coimbra-Costa *et al.,* 2017). Thus, frequent dives would lead to high levels of oxidative stress, can induce inflammation in for example the lungs and lead to compromised respiratory function. However, cetaceans do not exhibit any of these hypoxic effects (e.g. lung inflammation), suggesting an evolutionary drive for ‘hypoxia resistance’. Indeed, cetaceans lost the function of lung related genes involved in oxidative stress (e.g. MAP3K19) (Huelsmann *et al.,* 2019). The gene MAP3K19 can directly induce NF-ĸB transcription and lead to an inflammatory response (Boehme *et al.,* 2016). Therefore, the evolutionary changes we observed in the NF-ĸB pathway could also be linked to suppression of inflammation to cope with generation of reactive oxygen species induced by diving.

### Implications of energy metabolic adaptations for life-history functions

Finally, most components in the mTOR pathway were positively selected including both of its complexes (mTORC1 and mTORC2). mTORC1 regulates processes related to growth and differentiation while mTORC2 plays a regulatory role in the insulin cascade (Lamming *et al.,* 2012). As we observed changes in key genes involved in insulin signaling, it is maybe not surprising that several components in the mTOR pathway are significantly changed in cetaceans as well. mTORC1 plays a key role in sensing glucose to regulate glycolysis under nutrient-rich environments. Interestingly, muscle specific knockout of mTOR in mice showed a significantly decreased expression of the glucose transporter GLUT4 (Zhang *et al.,* 2014). Higher levels of circulating factor 4E binding proteins E and A (eIF4E and eIF4A) are associated with lowered risk of type-2 diabetes. Their predicted inhibition would further support attempts to maintain circulating glucose levels. Hence, similar to the insulin cascade, this would also suggest evolutionary adaptations related to prey with low glucose content. In addition, mTORC1 is also regulated via the hypoxia-inducible factor-1 (HIF-1), which is a transcription factor responsive to low oxygen levels (Li *et al.,* 2007). HIF-1 is also a regulator for oxygen independent glycolysis and suppresses the oxygen dependent oxidative phosphorylation in the mitochondria (Semenza, 2007). This might reflect the need to regulate glycolysis under hypoxic conditions, but pyruvate kinase (rate limited and last step in anaerobic glycolysis) does not seems to differ between marine and land mammals (Castellini *et al.,* 1981). The pentose phosphate pathway (shuttles the first compound of the glycolysis glucose-6-phosphate) may be of great importance to provide cells with glucose under oxygen limited environments, which is mediated by mTOR (Tsouko *et al.,* 2014). Hence, the observed changes to the mTOR complex do raise the prospect that energy investment decisions into body maintenance and growth, and therefore also reproduction, under varying nutrient environments differ from terrestrial counterparts (Papadopoli *et al.,* 2019). Indeed, p70S6K, which we found is significantly altered in cetaceans, is involved in mediating the post-fertilization embryo investment effects of mTOR inhibition (Guo and Yu, 2019). The potential interplay of these adaptations needs to be further investigated to understand how these adaptations will affect cetacean life history decisions under varying nutrient landscapes.

## Conclusion

Taken together, our findings provide novel insights into the role of the insulin, mTOR and NF-ĸB signaling pathways in the adaptation of cetaceans to an aquatic life. Our focus on biological pathway-level expected changes as adaptations to an aquatic lifestyle across the whole cetacean group has opened up novel and exciting research avenues into phenotypic and molecular evolution of particular cetacean taxa and candidate genes. Population genomics studies will be required to determine spatio-temporal structure of adaptive genetic diversity at key genes in nutrient sensing pathways. Our work has also provided a framework for examining physiological trait evolution throughout cetartiodactyla.

These results mean that condition measures based on adiposity must be used with caution. Blubber thickness alone provides little insight into the health of cetaceans (Kershaw *et al.,* 2019; Derous *et al.,* 2020). This is likely because blubber is a subcutaneous layer of fat and the evolutionary adaptation to marine life in these species lead to a thickening of that fat layer.

Lower bounds of adiposity are influenced by thermoregulatory requirements and upper bounds of adiposity will not be influenced by inflammatory response in the same way as it is in terrestrial mammals. They also point to adaptations to glucose-poor prey items that are distributed more heterogeneously than on land. These two novel pressures (thermoregulatory needs for adiposity and a novel prey field) have led to changes in energy metabolism, which may also affect the way energy investment decisions are reached in cetaceans. Blubber is a complex tissue (Kershaw *et al.,* 2018) and is significantly more metabolically active than subcutaneous fats in terrestrial mammals. Further work is needed to unravel the complex signaling mechanisms of adipose tissue in cetacean energy metabolism and to determine the effects of these signaling molecules on whole body functioning including appetite regulation, energy balance and inflammatory responses. Understanding these evolutionary adaptations to cetacean metabolism can help us move towards finding novel markers to assess the impact of stressors on cetacean health. These novel health markers can help us manage conservation threats that perturb the environmental nutrient levels of cetaceans.

## Authors’ contribution

D.L. and D.D. designed the study. D.D. and M.W. wrote the manuscript, and D.D. performed the pathway level analyses and interpretation with input from D.L. M.W. performed the gene-level analyses with input from A.D. and J.S. D.D., M.W. and D.L. interpreted the data. All authors read and commented on the manuscript.

## Data accessibility

Data will become openly available after uploading on Dryad.

## Supplementary Material

suppl_data_coaa136
