# Supplementary material for: Comparative genomics of cetartiodactyla: energy metabolism underpins the transition to an aquatic lifestyle
Source: Conserv Physiol. 2021 Jan 16;9(1):coaa136. doi: 10.1093/conphys/coaa136 (PMC7816800; doi:10.1093/conphys/coaa136)
Supplement: suppl_data_coaa136 [file suppl_data_coaa136.zip › Supplementary Tables.docx]

# **Supplementary Tables**

| **Table S1.** The genome assemblies of human, mouse, 16 cetacean species and 37 artiodactyl species downloaded from NCBI. | | |
| --- | --- | --- |
| **Group** | **Species** | **NCBI accession** |
| Artiodactyla | Ammotragus lervia | GCA_002201775.1 |
| Artiodactyla | Antilocapra americana | GCA_004027515.1 |
| Artiodactyla | Axis porcinus | GCA_003798545.1 |
| Artiodactyla | Beatragus hunteri | GCA_004027495.1 |
| Artiodactyla | Bison bison | GCA_000754665.1 |
| Artiodactyla | Bos indicus | GCA_000247795.2 |
| Artiodactyla | Bos mutus | GCA_000298355.1 |
| Artiodactyla | Bos taurus | GCA_002263795.2 |
| Artiodactyla | Bubalus bubalis | GCA_003121395.1 |
| Artiodactyla | Camelus bactrianus | GCA_000767855.1 |
| Artiodactyla | Camelus dromedarius | GCA_000767585.1 |
| Artiodactyla | Camelus ferus | GCA_000311805.2 |
| Artiodactyla | Capra aegagrus | GCA_000978405.1 |
| Artiodactyla | Capra hircus | GCA_001704415.1 |
| Artiodactyla | Capra sibirica | GCA_003182615.2 |
| Artiodactyla | Capreolus capreolus | GCA_000751575.1 |
| Artiodactyla | Catagonus wagneri | GCA_004024745.1 |
| Artiodactyla | Cervus elaphus | GCA_002197005.1 |
| Artiodactyla | Elaphurus davidianus | GCA_002443075.1 |
| Artiodactyla | Giraffa tippelskirchi | GCA_001651235.1 |
| Artiodactyla | Hemitragus hylocrius | GCA_004026825.1 |
| Artiodactyla | Hippopotamus amphibius | GCA_002995585.1 |
| Artiodactyla | Moschus moschiferus | GCA_004024705.1 |
| Artiodactyla | Odocoileus hemionus | GCA_004115125.1 |
| Artiodactyla | Odocoileus virginianus | GCA_002102435.1 |
| Artiodactyla | Okapia johnstoni | GCA_001660835.1 |
| Artiodactyla | Oryx gazella | GCA_003945745.1 |
| Artiodactyla | Ovis ammon | GCA_003121645.1 |
| Artiodactyla | Ovis aries | GCA_002742125.1 |
| Artiodactyla | Ovis canadensis | GCA_004026945.1 |
| Artiodactyla | Pantholops hodgsonii | GCA_000400835.1 |
| Artiodactyla | Pseudois nayaur | GCA_003182575.1 |
| Artiodactyla | Rangifer tarandus | GCA_004026565.1 |
| Artiodactyla | Saiga tatarica | GCA_004024985.1 |
| Artiodactyla | Sus scrofa | GCA_000003025.6 |
| Artiodactyla | Tragulus javanicus | GCA_004024965.1 |
| Artiodactyla | Vicugna pacos | GCA_000164845.3 |
| Cetacea | Balaenoptera acutorostrata | GCA_000493695.1 |
| Cetacea | Balaenoptera bonaerensis | GCA_000978805.1 |
| Cetacea | Delphinapterus leucas | GCA_002288925.2 |
| Cetacea | Eschrichtius robustus | GCA_002189225.1 |
| Cetacea | Lagenorhynchus obliquidens | GCA_003676395.1 |
| Cetacea | Lipotes vexillifer | GCA_000442215.1 |
| Cetacea | Megaptera novaeangliae | GCA_004329385.1 |
| Cetacea | Mesoplodon bidens | GCA_004027085.1 |
| Cetacea | Monodon monoceros | GCA_004027045.1 |
| Cetacea | Neophocaena asiaeorientalis | GCA_003031525.1 |
| Cetacea | Orcinus orca | GCA_000331955.2 |
| Cetacea | Phocoena phocoena | GCA_003071005.1 |
| Cetacea | Physeter catodon | GCA_002837175.2 |
| Cetacea | Sousa chinensis | GCA_003521335.2 |
| Cetacea | Tursiops aduncus | GCA_003227395.1 |
| Cetacea | Tursiops truncatus | GCA_001922835.1 |
| Outgroup | Homo sapiens | GCA_000001405.27 |
| Outgroup | Mus musculus | GCA_000001635.8 |

| **Table S2**. Insulin signaling pathway overlaid with the likelihood-ratio test statistics (2ΔL) between the positive and neutral branch-models as a measure to visualize a pathway effect. | | | |
| --- | --- | --- | --- |
| **Symbol** | **2ΔL** | ***p*-value** |  |
| 4E-BP1 | 0.000 | 1.000 |  |
| ACLY | 17.800 | <0.001 |  |
| AFX | 0.000 | 1.000 |  |
| AKT |  |  |  |
| AMP |  |  |  |
| Apoptosis |  |  |  |
| ATP |  |  |  |
| BAD | 1.359 | 0.566 |  |
| c-RAF | 105.387 | <0.001 |  |
| C3G | 0.156 | 1.000 |  |
| cAMP |  |  |  |
| CBL | 0.000 | 1.000 |  |
| Cell growth |  |  |  |
| CIP4 | 0.003 | 1.000 |  |
| CRK |  |  |  |
| eIF2B |  |  |  |
| eIF4E | 0.333 | 1.000 |  |
| ENAC |  |  |  |
| ER stress |  |  |  |
| ERK1/2 |  |  |  |
| Fatty acid synthesis |  |  |  |
| FKHR |  |  |  |
| FKHRL1 | 0.000 | 1.000 |  |
| FYN | 8.207 | 0.016 |  |
| GAB1 | 11.223 | 0.004 |  |
| Glucose |  |  |  |
| GLUT4 | 0.188 | 1.000 |  |
| GRB10 | 3.968 | 0.139 |  |
| GRB2 | 50.205 | <0.001 |  |
| GSK3 |  |  |  |
| GYS |  |  |  |
| INSR | 34.960 | <0.001 |  |
| INSULIN |  |  |  |
| IRS |  |  |  |
| IRS1 | 0.001 | 1.000 |  |
| JAK1/2 |  |  |  |
| JNK1 | 0.001 | 1.000 |  |
| LAR | 0.000 | 1.000 |  |
| LIPE | 16.254 | <0.001 |  |
| Lipolysis |  |  |  |
| MEK1/2 |  |  |  |
| mTOR | 41.010 | <0.001 |  |
| NCK | 9.400 | 0.009 |  |
| p70 S6K |  |  |  |
| PDE3B | 105.839 | <0.001 |  |
| PDK1 | 0.000 | 1.000 |  |
| PI3K |  |  |  |
| PIK3R1 | 22.707 | <0.001 |  |
| PIK3R2 | 20.576 | <0.001 |  |
| PIP2 |  |  |  |
| PIP3 |  |  |  |
| PKA |  |  |  |
| PKC(λ,ζ) |  |  |  |
| PP1 |  |  |  |
| Protein synthesis |  |  |  |
| PTEN | 0.019 | 1.000 |  |
| PTP1B | 14.851 | 0.001 |  |
| RAPTOR | 0.318 | 1.000 |  |
| RAS |  |  |  |
| SGK | 0.110 | 1.000 |  |
| SHC | 0.000 | 1.000 |  |
| SHIP |  |  |  |
| SHP2 | 9.205 | 0.009 |  |
| SOCS3 | 0.000 | 1.000 |  |
| Sodium transport |  |  |  |
| SOS |  |  |  |
| STX4 | 0.000 | 1.000 |  |
| SYNIP | 0.000 | 1.000 |  |
| TC10 | 31.276 | <0.001 |  |
| Transcription |  |  |  |
| TSC1 | 4.121 | 0.129 |  |
| Tsc1-Tsc2 |  |  |  |
| TSC2 | 31.417 | <0.001 |  |
| VAMP2 | 3.423 | 0.185 |  |

| **Table S3.** mTOR signaling pathway overlaid with the likelihood-ratio test statistics (2ΔL) between the positive and neutral branch-models as a measure to visualize a pathway effect. | | |
| --- | --- | --- |
| **Symbol** | **2ΔL** | ***p*-value** |
| 40S Ribosome-eIF3-mRNA-eIF4A-eIF4B-eIF4E-eIF4G |  |  |
| 40SRibosome |  |  |
| 4EBP | 0.000 | 1.000 |
| 4EBP-eIF4E |  |  |
| Actin organization |  |  |
| AKT |  |  |
| AMP |  |  |
| AMPK |  |  |
| ATG13 | 24.238 | <0.001 |
| Autophagy regulation |  |  |
| DAG |  |  |
| DGKζ | 4.209 | 0.124 |
| eIF3 |  |  |
| eIF4A |  |  |
| eIF4A-eIF4B-eIF4E-eIF4G |  |  |
| eIF4B | 232.205 | <0.001 |
| eIF4E | 0.333 | 1.000 |
| eIF4G |  |  |
| ERK1/2 |  |  |
| FKBP1 | 0.010 | 1.000 |
| GBL | 20.882 | <0.001 |
| HIF1α | 0.000 | 1.000 |
| Hypoxia |  |  |
| INSR | 34.960 | <0.001 |
| INSULIN |  |  |
| IRS1 | 0.001 | 1.000 |
| LKB1 | 11.489 | 0.003 |
| mTOR | 41.010 | <0.001 |
| mTORC1 |  |  |
| mTORC2 |  |  |
| Neurodegenerative diseases |  |  |
| p70S6K | 292.275 | <0.001 |
| PA |  |  |
| PC |  |  |
| PDK1 | 0.000 | 1.000 |
| PI3K |  |  |
| PIP2 |  |  |
| PIP3 |  |  |
| PKC |  |  |
| PKCα | 383.006 | <0.001 |
| PLD |  |  |
| PMA |  |  |
| PP2A |  |  |
| PRAS40 | 0.000 | 1.000 |
| PROTOR |  |  |
| RAC | 3.675 | 0.161 |
| Rapamycin |  |  |
| RAPTOR | 0.318 | 1.000 |
| RAS |  |  |
| REDD1 | 0.000 | 1.000 |
| RHEB | 42.036 | <0.001 |
| RHO |  |  |
| RICTOR | 472.142 | <0.001 |
| RPS6 | 1.495 | 0.531 |
| RSK |  |  |
| SIN1 | 10.430 | 0.005 |
| Translation |  |  |
| TSC1 | 4.121 | 0.129 |
| Tsc1-Tsc2 |  |  |
| TSC2 | 31.417 | <0.001 |
| ULK1 | 0.000 | 1.000 |
| VEGF |  |  |

| **Table S4.** NF-ĸB signaling pathway overlaid with the likelihood-ratio test statistics (2ΔL) between the positive and neutral branch-models as a measure to visualize a pathway effect. | | |
| --- | --- | --- |
| **Symbol** | **2ΔL** | ***p*-value** |
| β-TrCP | 77.820 | <0.001 |
| A20 | 0.000 | 1.000 |
| ABIN-1 | 4.063 | 0.132 |
| AKT |  |  |
| B-cell maturation |  |  |
| BAFF | 5.391 | 0.066 |
| Bcl10 | 0.000 | 1.000 |
| Bcl10-Card10-Malt1 |  |  |
| BIMP1 | 4.177 | 0.125 |
| BMP2/4 |  |  |
| BR3 | 5.818 | 0.053 |
| CARD11 | 1.116 | 0.639 |
| Caspase8 | 0.904 | 0.723 |
| CBP/p300 |  |  |
| CD40 | 24.403 | <0.001 |
| CD40L | 11.317 | 0.004 |
| Cell proliferation |  |  |
| Cell survival |  |  |
| Chuk-Ikbkb-Ikbkg |  |  |
| CK2 |  |  |
| Cot | 0.624 | 0.876 |
| EGF | 3.083 | 0.219 |
| FADD | 0.000 | 1.000 |
| GH | 0.000 | 1.000 |
| Growth factor receptor |  |  |
| GSK-3β | 55.087 | <0.001 |
| HDAC1/2 |  |  |
| IκB |  |  |
| IkB-NfkB1-RelA |  |  |
| IkB-NfkB2-RelA |  |  |
| IKKα | 129.661 | <0.001 |
| IKKβ | 0.014 | 1.000 |
| IKKγ | 2.158 | 0.368 |
| IL-1 |  |  |
| IL-1R/TLR |  |  |
| Immune response |  |  |
| Inflammation |  |  |
| Insulin |  |  |
| IRAK1/4 |  |  |
| IRAK-M | 0.000 | 1.000 |
| JNK1 | 0.001 | 1.000 |
| LCK | 0.000 | 1.000 |
| LTA | 1.123 | 0.639 |
| LTBR | 570.904 | <0.001 |
| Lymphogenesis |  |  |
| MALT1 | 0.000 | 1.000 |
| MEKK1 | 0.000 | 1.000 |
| MEKK3/NIK |  |  |
| MKK6/7 |  |  |
| MYD88 | 1.355 | 0.566 |
| NAK | 0.023 | 1.000 |
| NAP1 | 0.208 | 1.000 |
| NF-κB p50/p52 |  |  |
| NF-κB1 | 0.000 | 1.000 |
| NF-κB2 p100 | 0.000 | 1.000 |
| NfkB-RelA |  |  |
| NfkB1-RelA |  |  |
| NfkB2(p52)-RelB |  |  |
| NGF | 0.145 | 1.000 |
| NIK |  |  |
| p65/RelA | 0.000 | 1.000 |
| PELI1 | 0.000 | 1.000 |
| PI3K |  |  |
| PKAc |  |  |
| PKC(β,θ) |  |  |
| PKCζ | 31.135 | <0.001 |
| PKR | 0.000 | 1.000 |
| PLCγ2 | 0.000 | 1.000 |
| Raf |  |  |
| RANKL | 1.048 | 0.670 |
| Ras |  |  |
| RelB | 0.500 | 0.945 |
| RIP | 5.280 | 0.070 |
| TAB1 | 9.539 | 0.008 |
| TAB2/3 |  |  |
| TAK1 | 1.581 | 0.504 |
| TANK | 8.987 | 0.010 |
| TCR |  |  |
| TGF-α | 1.477 | 0.535 |
| TIRAP | 0.000 | 1.000 |
| TNF-α | 0.420 | 1.000 |
| TNFR |  |  |
| TRADD | 2.123 | 0.375 |
| TRAF2/3/5 |  |  |
| TRAF5/6 |  |  |
| TRAF2 | 0.000 | 1.000 |
| TRAF6 | 1.795 | 0.444 |
| TTRAP | 0.000 | 1.000 |
| UBE2N | 0.011 | 1.000 |
| Ube2n-Ube2v1 |  |  |
| UBE2V1 | 0.000 | 1.000 |
| Zap70 | 11.445 | 0.003 |

| **Table S5**. SIRT signaling pathway overlaid with the likelihood-ratio test statistics (2ΔL) between the positive and neutral branch-models as a measure to visualize a pathway effect. | | |
| --- | --- | --- |
| **Symbol** | **2ΔL** | ***p*-value** |
| ACADL | 0.000 | 1.000 |
| ACSS1 | 0.260 | 1.000 |
| Alpha tubulin |  |  |
| Alzheimer disease |  |  |
| Apaf1-Cycs |  |  |
| ARNTL | 3.815 | 0.149 |
| Biogenesis of mitochondria |  |  |
| BIRC5 | 7.876 | 0.019 |
| Cancers and Tumors |  |  |
| CDKN1A | 0.000 | 1.000 |
| Cell death |  |  |
| Cell survival |  |  |
| Circadian rhythm |  |  |
| CPS1 |  |  |
| CRTC2 | 0.001 | 1.000 |
| CTNNB1 | 0.001 | 1.000 |
| CYC1 | 0.000 | 1.000 |
| CYCS | 0.000 | 1.000 |
| Cyct |  |  |
| cytochrome C |  |  |
| DNA damage |  |  |
| E2F1 | 1.341 | 0.566 |
| EPAS1 | 0.001 | 1.000 |
| Feeding |  |  |
| Foxo |  |  |
| FOXO1 | 0.000 | 1.000 |
| FOXO3 | 0.000 | 1.000 |
| FOXO4 | 0.000 | 1.000 |
| FOXO6 | 0.000 | 1.000 |
| Gluconeogenesis |  |  |
| GLUD1 | 6.982 | 0.030 |
| HCRTR2 | 0.000 | 1.000 |
| HIF1A | 0.000 | 1.000 |
| HSF1 | 0.000 | 1.000 |
| Hypoxia |  |  |
| IDE | 0.000 | 1.000 |
| IDH2 | 0.000 | 1.000 |
| Inflammation |  |  |
| Insulin sensitivity |  |  |
| Memory |  |  |
| MRPL10 | 0.049 | 1.000 |
| NDUFA9 | 0.000 | 1.000 |
| NFkB (complex) |  |  |
| NFKB1 | 0.000 | 1.000 |
| NFKB2 | 0.000 | 1.000 |
| NR1H2 | 0.000 | 1.000 |
| NR1H3 | 0.000 | 1.000 |
| Oxidation of fatty acid |  |  |
| PARP1 | 22.597 | <0.001 |
| PER2 | 0.000 | 1.000 |
| PIP5K1A | 0.000 | 1.000 |
| PIP5K1C | 5.059 | 0.078 |
| PPARA | 17.202 | <0.001 |
| PPARG | 0.000 | 1.000 |
| PPARGC1A | 0.164 | 1.000 |
| PPID | 33.429 | <0.001 |
| RARA | 0.287 | 1.000 |
| RARB | 242.778 | <0.001 |
| Rb |  |  |
| RB1 | 61.629 | <0.001 |
| RBL1 | 10.260 | 0.006 |
| RBL2 | 53.656 | <0.001 |
| RELA | 0.000 | 1.000 |
| SDHA | 2.223 | 0.354 |
| SDHB | 14.946 | 0.001 |
| SIRT1 | 161.940 | <0.001 |
| SIRT2 | 0.001 | 1.000 |
| SIRT3 | 6.500 | 0.038 |
| SIRT4 | 0.282 | 1.000 |
| SIRT5 | 0.000 | 1.000 |
| SIRT6 | 8.713 | 0.012 |
| SIRT7 | 0.000 | 1.000 |
| SLC25A5 | 2.810 | 0.254 |
| SLC25A6 | 87.909 | <0.001 |
| SMAD7 | 64.800 | <0.001 |
| SREBF1 | 4.267 | 0.120 |
| SREBF2 | 0.935 | 0.714 |
| Suppression of tumor |  |  |
| TLE1 | 0.000 | 1.000 |
| TP53 | 0.000 | 1.000 |
| TSC2 | 31.417 | <0.001 |
| TUBA1A | 4.839 | 0.088 |
| TUBA1B | 0.000 | 1.000 |
| TUBA1C | 25.230 | <0.001 |
| TUBA3C/TUBA3D | 11.106 | 0.004 |
| TUBA3E | 10.283 | 0.006 |
| TUBA4A | 0.000 | 1.000 |
| TUBA4B |  |  |
| TUBA8 | 0.027 | 1.000 |
| UCP2 | 0.000 | 1.000 |
| WRN | 6.106 | 0.046 |
| XRCC6 | 0.119 | 1.000 |

| **Table S6.** P53 signaling pathway overlaid with the likelihood-ratio test statistics (2ΔL) between the positive and neutral branch-models as a measure to visualize a pathway effect. | | |
| --- | --- | --- |
| **Symbol** | **2ΔL** | ***p*-value** |
| 14-3-3σ | 0.000 | 1.000 |
| AKT |  |  |
| Angiogenesis |  |  |
| Apaf1 | 1.169 | 0.625 |
| Apoptosis |  |  |
| ASPP |  |  |
| ATM | 2.078 | 0.382 |
| ATR | 0.065 | 1.000 |
| Autophagy |  |  |
| BAI1 | 2.103 | 0.378 |
| BAX | 0.000 | 1.000 |
| Bcl-2 | 0.077 | 1.000 |
| Bcl-xL | 0.000 | 1.000 |
| Brca1 |  |  |
| CABC1 |  |  |
| Caspase 6 |  |  |
| CDK2 | 0.347 | 1.000 |
| CDK2-Cyclin D1 |  |  |
| CDK4 | 0.000 | 1.000 |
| CDK4-Cyclin D2 |  |  |
| Cell cycle arrest |  |  |
| Cell cycle progression |  |  |
| Cell survival |  |  |
| Chk1 | 0.000 | 1.000 |
| Chk2 | 0.001 | 1.000 |
| c-Jun | 2.366 | 0.327 |
| CK1δ |  |  |
| Cyclin D1 | 0.014 | 1.000 |
| Cyclin D2 | 0.010 | 1.000 |
| CyclinG | 13.155 | 0.001 |
| CyclinK |  |  |
| DNA damage |  |  |
| DNA repair |  |  |
| DNA-PK |  |  |
| DR4/5 |  |  |
| DRAM |  |  |
| E2F1 | 1.341 | 0.566 |
| E2f1-Rb |  |  |
| FAS | 1.763 | 0.450 |
| GADD45 |  |  |
| Glycolysis |  |  |
| GML |  |  |
| Gsk3β | 55.087 | <0.001 |
| HDAC |  |  |
| HDAC9 |  |  |
| HIF1A | 0.000 | 1.000 |
| HIPK2 |  |  |
| Hypoxia |  |  |
| JMY |  |  |
| Jmy-p300 |  |  |
| JNK1 | 0.001 | 1.000 |
| Maspin | 0.223 | 1.000 |
| MDM2 | 0.000 | 1.000 |
| MDM4 | 0.001 | 1.000 |
| Mitochondrial respiration |  |  |
| NOXA | 9.495 | 0.008 |
| Nucleostemin |  |  |
| p19arf | 23.352 | <0.001 |
| p21Cip1 | 0.000 | 1.000 |
| p300 |  |  |
| p38 MAPK |  |  |
| p48 |  |  |
| p53 | 0.000 | 1.000 |
| p53AIP1 |  |  |
| p53R2 | 0.018 | 1.000 |
| p63 |  |  |
| p73 | 0.564 | 0.915 |
| PAI-1 |  |  |
| PCAF |  |  |
| PCNA |  |  |
| PERP | 22.105 | <0.001 |
| PI3K |  |  |
| PIAS1 |  |  |
| PIDD | 0.001 | 1.000 |
| PIG3 | 18.848 | <0.001 |
| PML |  |  |
| PTEN | 0.019 | 1.000 |
| PUMA | 33.998 | <0.001 |
| Rb | 61.629 | <0.001 |
| Reprimo | 0.000 | 1.000 |
| SCO2 |  |  |
| Senescence |  |  |
| SIRT | 161.940 | <0.001 |
| Slug |  |  |
| STAG1 |  |  |
| Survivin | 7.876 | 0.019 |
| Teap |  |  |
| TIGAR |  |  |
| TOPBP1 |  |  |
| TRAP220 |  |  |
| TRIM29 |  |  |
| TSP1 | 0.016 | 1.000 |
| Tumor suppression |  |  |
| UCN-01 |  |  |
| WT1 |  |  |
| ZAC1 |  |  |
| β-catenin | 0.001 | 1.000 |

| **Table S7.** Insulin signaling pathway overlaid with the likelihood-ratio test statistics (2ΔL) between the positive and neutral branch-models as a measure to visualize a pathway effect. | | |
| --- | --- | --- |
| **Symbol** | **2ΔL** | ***p*-value** |
| AKT |  |  |
| BCL-XL | 0.000 | 1.000 |
| c-FOS | 0.000 | 1.000 |
| c-JUN | 2.366 | 0.327 |
| c-Raf | 105.387 | <0.001 |
| CCK2R | 0.000 | 1.000 |
| CEBPβ | 0.000 | 1.000 |
| Cell proliferation |  |  |
| CIS | 0.000 | 1.000 |
| ERK1/2 |  |  |
| Gαq | 0.000 | 1.000 |
| GAST | 2.472 | 0.311 |
| GRB2 | 50.205 | <0.001 |
| IL-6 | 48.957 | <0.001 |
| JAK |  |  |
| JAK2 | 0.043 | 1.000 |
| LEP | 0.000 | 1.000 |
| LEPR | 0.000 | 1.000 |
| MEK1/2 |  |  |
| mTOR | 41.010 | <0.001 |
| NFκB |  |  |
| p21Cip1 | 0.000 | 1.000 |
| PI3K |  |  |
| PIAS |  |  |
| PTP1B | 14.851 | 0.001 |
| Ras |  |  |
| SHC | 0.000 | 1.000 |
| SHP1 | 0.000 | 1.000 |
| SHP2 | 9.205 | 0.009 |
| SOCS |  |  |
| SOS |  |  |
| STAT |  |  |
| Stat dimer |  |  |
| STAT3 | 2.391 | 0.323 |
| Stat3 dimer |  |  |
