# Supplementary figures and images for: Comparative genomics of cetartiodactyla: energy metabolism underpins the transition to an aquatic lifestyle
Source: Conserv Physiol. 2021 Jan 16;9(1):coaa136. doi: 10.1093/conphys/coaa136 (PMC7816800; doi:10.1093/conphys/coaa136)

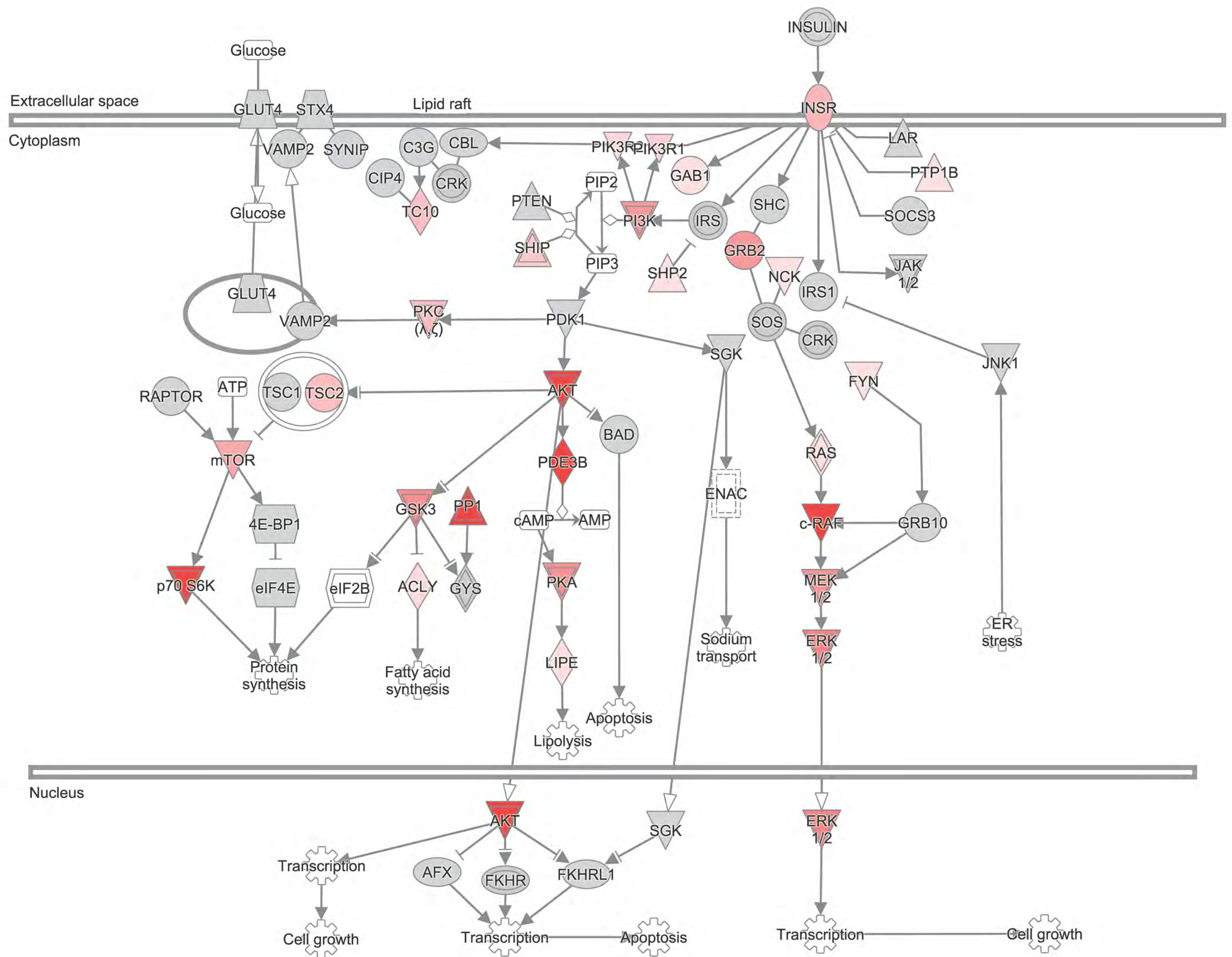

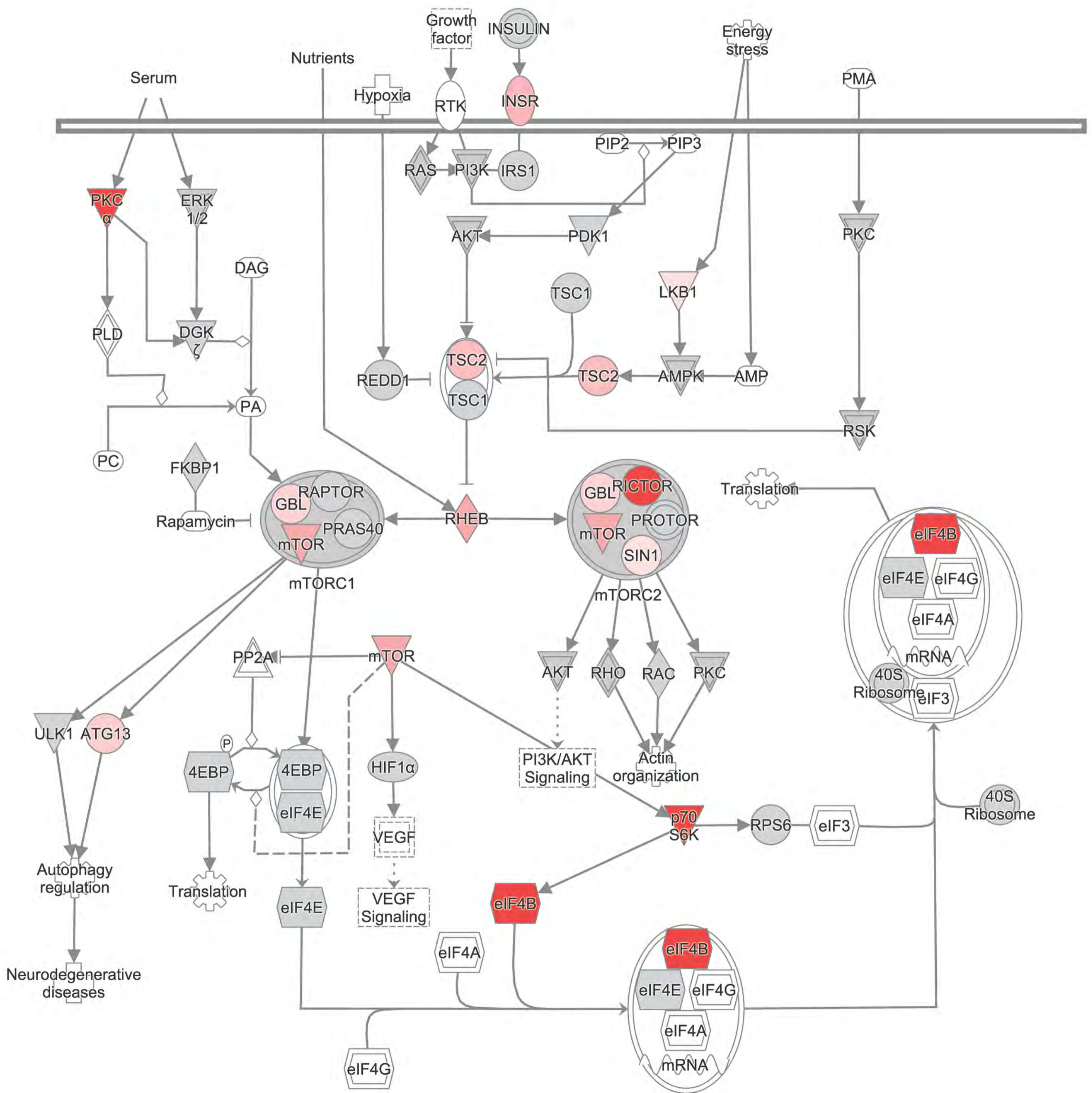

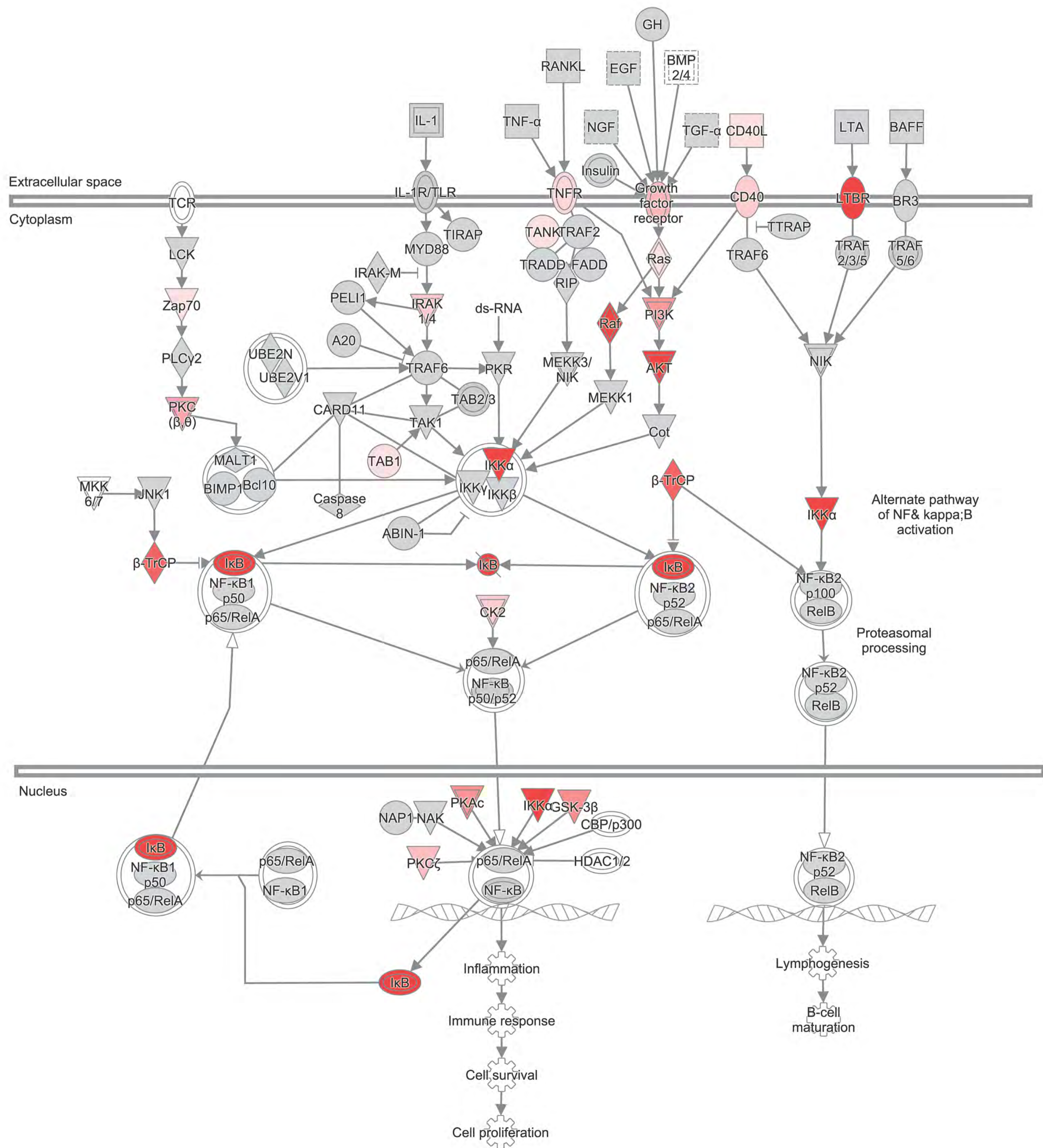

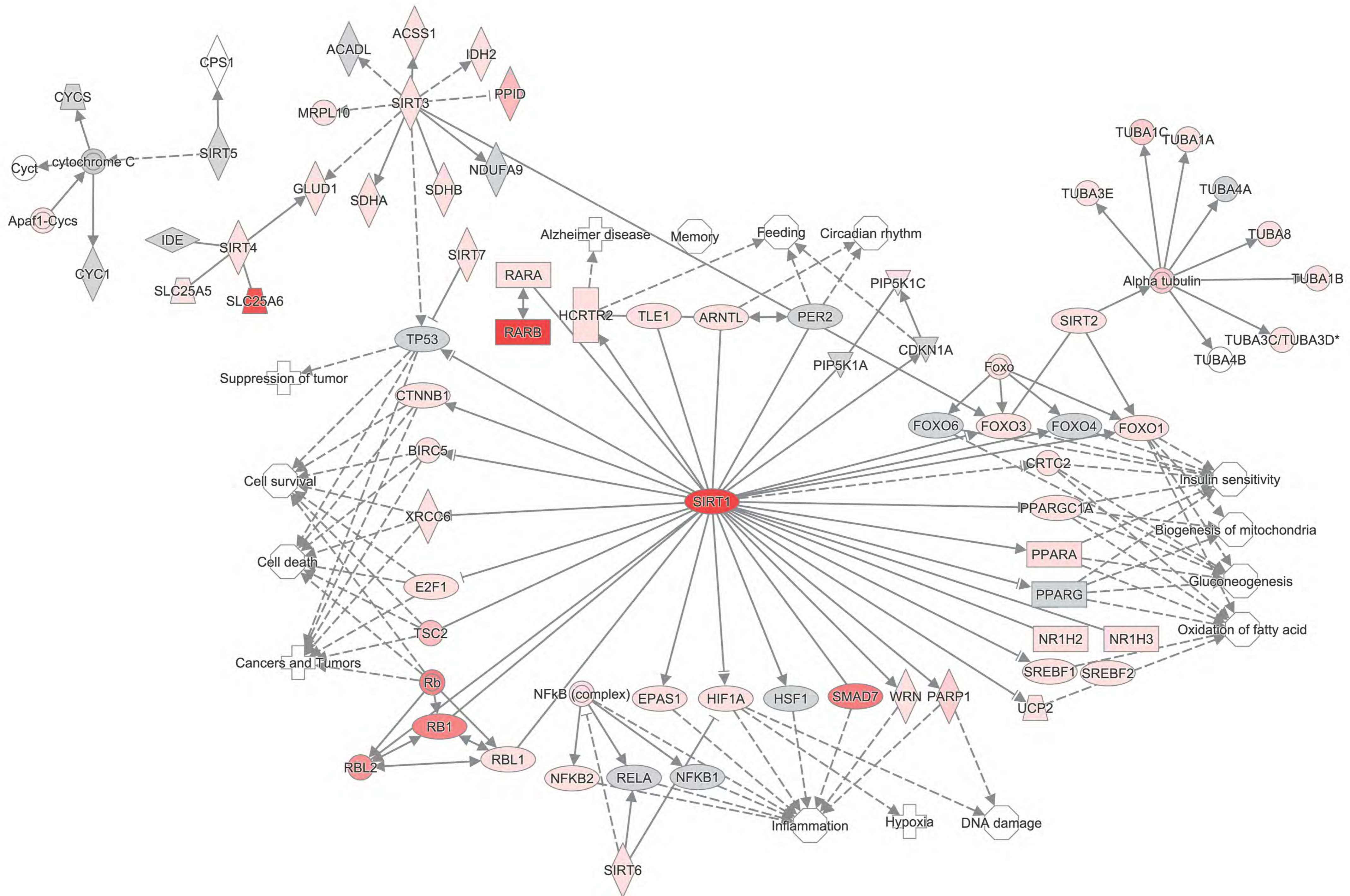

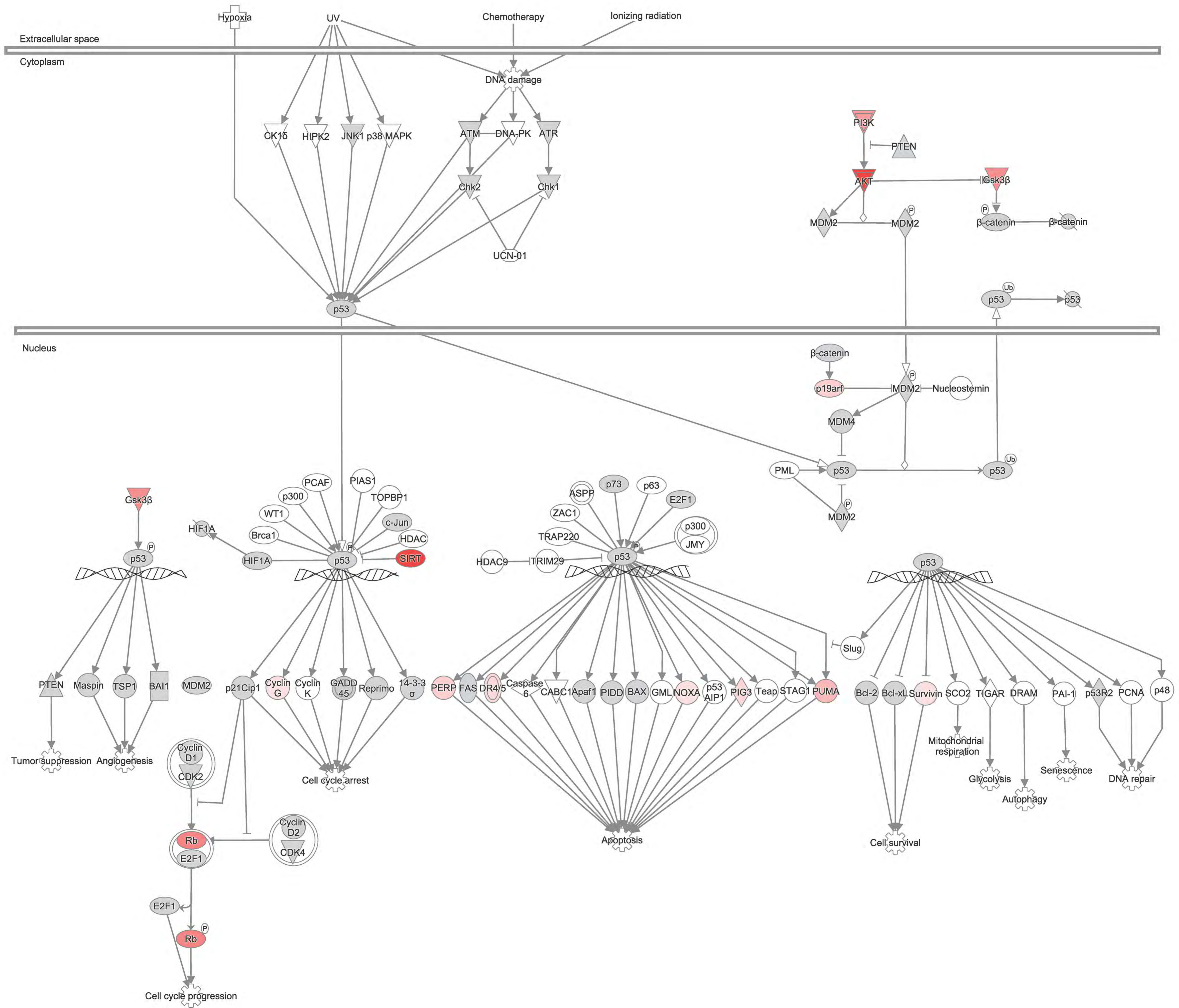

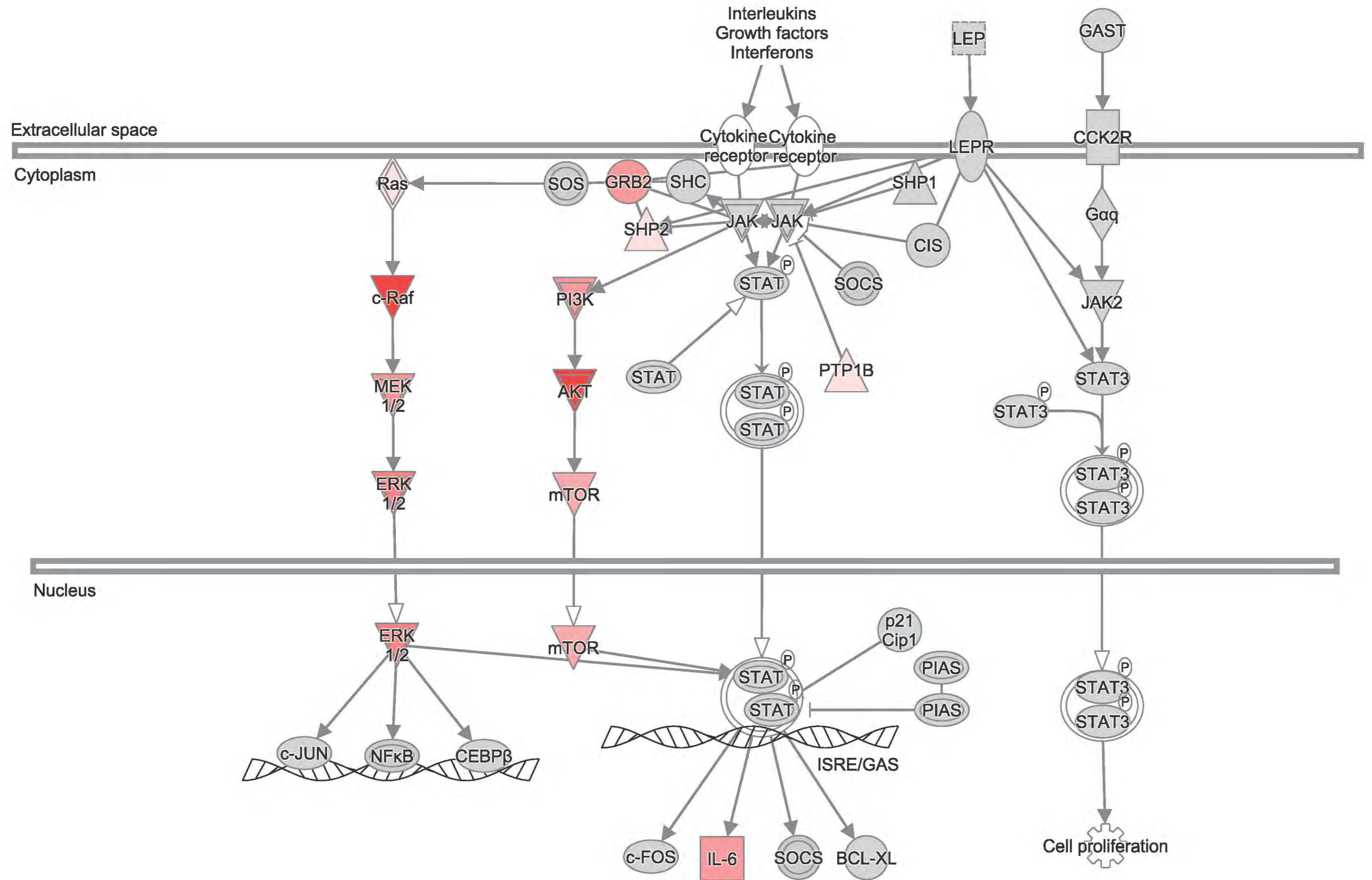

Supplement: suppl_data_coaa136 [file suppl_data_coaa136.zip › Supplementary figures.pdf]
